# Healthcare Experiences Are Associated with Colorectal Cancer Mortality but only for Specific Racial Groups: a SEER-CAHPS Study

**DOI:** 10.1007/s40615-023-01690-7

**Published:** 2023-06-27

**Authors:** Carol Y. Ochoa-Dominguez, Trevor A. Pickering, Stephanie Navarro, Claudia Rodriguez, Albert J. Farias

**Affiliations:** 1 Department of Population and Public Health Sciences, Keck School of Medicine of the University of Southern California, 2001 N. Soto St., Suite 318B, Los Angeles, CA 90032 USA; 2 Department of Radiation Medicine and Applied Sciences, University of California San Diego, San Diego, CA USA; 3 Dornsife College of Letters, Arts, and Sciences, University of Southern California, Los Angeles, CA USA; 4 Gehr Family Center for Health System Science, Keck School of Medicine of the University of Southern California, Los Angeles, CA USA

**Keywords:** Health disprities, Patient experiences with care, Quality of care

## Abstract

**Background:**

The objective of this study was to determine whether racial/ethnic disparities exist in patient-reported experiences with care after colorectal cancer diagnosis and whether they are associated with mortality.

**Methods:**

We conducted a retrospective cohort study of colorectal cancer patients diagnosed from 1997 to 2011, ≥ 65 years, and completed a Consumer Assessment of Healthcare Providers and Systems (CAHPS) survey at least 6 months after a cancer diagnosis. We leverage the National Cancer Institute’s SEER-CAHPS dataset of Medicare beneficiaries. CAHPS survey responses were used to generate four composite measures of patient experiences with 1) getting needed care, 2) getting needed prescription drugs, 3) getting care quickly, and 4) physician communication. We used multivariable linear regression models to examine racial differences in patient experiences with aspects of their care and multivariable Cox proportional hazards models to identify the risk of mortality associated with each composite score by racial group.

**Results:**

Of the 5135 patients, 76.86% were non-Hispanic White, 7.58% non-Hispanic Black, 8.30% Hispanic, and 7.26% non-Hispanic Asian. Overall, patients reported the highest scores for composite measures regarding “getting all needed prescriptions” and the lowest score for “getting care quickly.” In our adjusted models, we found that Hispanics, non-Hispanic Black, and non-Hispanic Asian patients reported significantly lower scores for getting needed prescription drugs (*B* = − 4.34, *B* = − 4.32, *B* = − 5.66; all *p* < 0.001) compared to non-Hispanic Whites. Moreover, non-Hispanic Black patients also reported lower scores for getting care quickly (*B* = − 3.44, *p* < 0.05). We only found one statistically significant association between composite scores of patient experience and mortality. For non-Hispanic Black patients, a 3-unit increase in getting needed care was associated with 0.97 times the hazard of mortality (*p* = 0.003).

**Conclusion:**

Our research underscores that CAHPS patient experiences with care are an important patient-centered quality-of-care metric that may be associated with cancer outcomes and that there may be differences in these relationships by race and ethnicity. Thus, highlighting how patients’ perceptions of their healthcare experiences can contribute to disparities in colorectal cancer outcomes.

## Background

Non-Hispanic Black (NHB) have the highest incidence of colorectal cancer (CRC) and have about a 40% increased risk of death due to CRC compared to non-Hispanic White (NHW) patients [1]. Overall, Hispanic and non-Hispanic Asians both have lower incidence and mortality rates than NHW, but disaggregated data has shown that Native Hawaiian and Pacific Islander men have about 26% increased rates of death than NHW men [2]. Multiple factors contribute to racial disparities in CRC survival, including sex, stage of diagnosis, tumor characteristics, hospital characteristics, socioeconomic status, and the presence of comorbidities [3, 4]. For instance, NHB colorectal cancer patients are less likely to undergo surgery [5, 6], receive radiation and chemotherapy [6], and initiate newer chemotherapy treatments [7] compared to NHW patients. Similarly, Hispanic colorectal patients are less likely to receive surgery, while non-Hispanic Asian colorectal patients are less likely to receive radiation than White patients; this has been associated with low socioeconomic status and physician-level variation [6].

Another driver of racial disparities in cancer treatment could be differences in patient experiences with medical care. These patient experiences with care are associated with colorectal cancer treatment and health outcomes. For instance, better global ratings of care (personal doctors and specialist doctors), the ability to get care quickly, and physician communication are associated with better general and mental health status [8], an earlier stage of CRC stage at diagnosis[9], receipt of colorectal cancer screening [10], and adherence to surveillance guidelines for colorectal cancer survivors [11]. High ratings for all global measures are associated with better general health status among breast, colorectal, lung, and prostate cancer survivors [12], as well as patients with cancer during the last year of their life [8]. Additionally, patient-provider communication is associated with double the likelihood of being screened for CRC [10]. Overall ratings of patients’ personal doctor and specialists is positively associated with adherence to office visits for surveillance guidelines among Medicare Fee-For-Service beneficiaries with colorectal cancer [11]. Poor patient experiences with medical care may strongly influence cancer survivorship care; however, these studies do not examine the association between racial/ethnic disparities in patient experiences with care or cancer health outcomes.

Racial disparities in patient experiences with medical care are well documented [13–19], but limited work has examined the relationships between care experiences and mortality risk for cancer patients. Specifically, the focus on patient experiences has previously been used as merely a metric of healthcare quality and the existing gap in the literature has been linking patient experiences with health outcomes and exploring if there are differences based on race/ethnicity.

In a recent systematic review, several studies have found significant associations between Consumer Assessment of Healthcare Providers and Systems (CAHPS) patient experiences with care measures and health outcomes, suggesting that patient experiences may influence subsequent follow-up treatment and outcomes [20]. Two studies among cancer survivors (with various cancer types) have found that getting needed care, care coordination, and global ratings of their specialist physician and prescription drug plan are associated with a decrease mortality risk [21, 22]. A third study found no association between patient experiences and mortality among patients undergoing surgery for urologic malignancies [23]. Of these three studies, only one looked at these relationships by race/ethnicity among a cohort of lung cancer survivors, which demonstrated that among Hispanic cancer survivors, better patient experiences were associated with a higher mortality risk [22]. Therefore, the objective of our study was to examine whether there are racial/ethnic disparities in patient experiences with care among individuals diagnosed with colorectal cancer and to determine whether these disparities are associated with colorectal cancer mortality.

## Methods

### Data Source

We used data from the National Cancer Institute’s Surveillance, Epidemiology, and End Results-Consumer Assessment of Healthcare Providers and Systems (SEER-CAHPS®), which links three secondary data sources: 1) SEER cancer registry data, 2) Centers for Medicare and Medicaid Services (CMS) CAHPS surveys, and 3) administrative or billing data through Medicare claims and enrollment records [24].

The SEER data contains demographic and tumor information on patients diagnosed with cancer within SEER regions throughout the USA. CMS data included claims from physicians and inpatient and outpatient facilities, which represents about 28% of the US population. The Consumer Assessment of Healthcare Providers and Systems (CAHPS) survey of patient experiences is a national probability-sample survey conducted through phone and email of Medicare beneficiaries that assesses various measures relating to perceived quality of and access to care. The majority of the sample completed the CAHPS survey methods via phone; however, there was a range between 70 and 89% based on race/ethnicity. Lastly, the Medicare claims and enrollment record include the assessment of health service use and mortality.

### Study Sample

We used a procedure similar to other published studies using SEER-CAHPS surveillance data [8, 11]. This study sample included individuals who were 1) 65 years of age or older at diagnosis, 2) were diagnosed with colon or rectal adenocarcinoma (International Classification of Diseases for Oncology, Third Edition codes C180, C182–C189, C199, C209) as a single, first primary cancer between 1997 and 2011, and 3) submitted a CAHPS survey at least 6 months after a cancer diagnosis. When an individual submitted multiple CAHPS surveys, we used only the first survey closest to cancer diagnosis for analysis. Of note, the differences in sample sizes in the subsequent analyses are due to certain CAHPS measures being introduced at various years [24].

### Measures

#### Patient Race/Ethnicity

Our primary independent variable for this analysis was race/ethnicity. We created a mutually exclusive variable using self-reported data from the CAHPS survey, when missing, the race variable from Medicare SEER was used. This variable had four categories: non-Hispanic White, non-Hispanic Black, Hispanic, and non-Hispanic Asian. Specifically, if an individual classified as Hispanic in any of the surveys they were categorized as Hispanic. Whereas all other individuals who classified as Asian/Pacific Islander, Black, or White were categorized as non-Hispanic.

#### Patient Experience of Care

Variables assessing patients’ quality of care included patient ratings of care and composite scores of patients’ experiences with care. Five composite scores of patient experiences in CAHPS were assessed on a 100-point scale and include getting all needed care (2 items), getting care quickly (2 items), quality of physician communication (4 items), health plan customer service (2 items), and access to prescriptions (1 item). Global ratings of care were assessed on a 10-point scale and included one question per item: rating their health plan, their health care, their primary physician, their specialist, and their prescription drug plan. Questions can be found in Ref. [25]. Following NIH recommendations, we examined the five composite scores using the linear mean scoring method to measure healthcare experiences [26].

#### Covariates

Demographic information was collected from all three data sources (SEER, CMS, and CAHPS data sets). In some instances, variables were constructed from multiple data sets to reduce missingness (e.g., race/ethnicity). We adjusted for demographic variables including age at survey, gender (female or male), marital status (married, non-married, or unknown), the poverty level of the census tract in which the patient resided (0 to 5%, 5 to < 10%, 10 to < 20%, 20 to 100%, or missing), educational attainment (high school or less, at least some college and higher, or missing), and health plan type (MA PDP, MA only, FFS PDP, FFS only, or MA PPO). Additionally, state of residence was recoded into geographical regions (West, South, Midwest, Northeast) using the divisions in the US Census Bureau [27]. For health variables we adjusted for the number of comorbid conditions which we use the NCI comorbidly index. The NCI comorbidity index is a modified Charlson comorbidity index with 14 chronic conditions. It has been modified to exclude solid tumors given that it was developed from a cohort of cancer patients. Cancer history variables included months from diagnosis to survey, stage of primary cancer (0, I, II, III, or IV), having undergone surgery as part of cancer treatment (yes, no, or missing), and having undergone radiation treatment (yes, no, or missing). We also adjusted for mode of the survey (mail, phone, or missing).

#### Mortality

Mortality status was measured through the end of 2013. The date of patient death was taken from Medicare records indicating the date the patient died or an indicator of whether the patient was still alive.

### Statistical Analysis

Descriptive and bivariate analyses were performed overall and stratifying the sample by race. Specifically, we used a Pearson’s chi-square to examine the association between categorical variables and race, and the Kruskal-Wallis test to examine the relationship between continuous variables (i.e., time from diagnosis to survey date) and race. Additionally, we examined the means of the quality of care composite variables and patient ratings by the levels of demographic variables using the Wilcoxon rank sum test (when there were only two categories) or the Kruskal-Wallis test (when there are more than two categories). To account for multiple comparisons between the various demographic variables and the 8 different outcomes and to

minimize the chance of type 1 error, we used the Benjamini-Hochberg method. Multivariable linear regression models were used to assess the association between patient race/ethnicity and composite scores, adjusting for the covariates mentioned above.

Cox proportional hazards models were used to determine the association between patient composite scores and mortality after a cancer diagnosis. To determine if the effect of these composite scores on mortality differed by race, a score-by-race interaction term was included in the models. We also stratified these models by survey date (median cut point of 42 months) to examine whether the time between cancer diagnosis and survey moderated model effects. All analyses were performed in R v3.4.4 in which we used tests for significance that were two sided and assessed at a significance level of *p* < 0.05 [28].

## Results

### Demographics, Covariates, and Race

Demographic characteristics of the overall sample and stratified by race are presented in Table 1. The majority of the 5135 patients retained for the analytic sample were NHW(76.9%), with 7.6% NHB, 8.3% Hispanic, and 7.3% non-Hispanic Asian. Patients tended to be more female (54.3%) and married (57.7%). Overall, patients were on average 74.7 (SD = 6.6) years old at diagnosis and 79.7 (SD = 6.8) years old at the time of the first survey after diagnosis. Most patients were diagnosed with stage II/III cancer (51.2%) and had no reported comorbidities (65.7%). The year of survey completion ranged from 2000 to 2013.

**Table 1:** Distribution of sociodemographic characteristics for patients diagnosed with colorectal cancer^1^

	Overall, *N* = 5135^2^	Non-Hispanic White, *N* = 3497^2^	Non-Hispanic Black, *N* = 389^2^	Hispanic, *N* = 426^2^	Non-Hispanic Asian, *N* = 373^2^	*p*-value^3^
Gender						<0.001
Female	2790 (54.3)	2100 (53.2)	272 (69.9)	213 (50.0)	205 (55.0)	
Male	2345 (45.7)	1847 (46.8)	117 (30.1)	213 (50.0)	168 (45.0)	
Age at survey						<0.001
65–70	335 (6.9)	242 (6.1)	43 (11.1)	34 (8.0)	34 (9.1)	
70–75	1096 (21.3)	807 (20.4)	102 (26.2)	102 (23.9)	85 (22.8)	
75–80	1308 (25.5)	995 (25.2)	116 (29.8)	111 (26.1)	86 (23.1)	
80–85	1182 (23.0)	938 (23.8)	69 (17.7)	95 (22.3)	80 (21.4)	
85 or older	1196 (23.3)	965 (24.4)	59 (15.2)	84 (19.7)	88 (23.6)	
Marital status at diagnosis						<0.001
Non-married	1934 (37.7)	1423 (36.1)	228 (58.6)	169 (39.7)	> 113 (> 30.3)	
Married	2965 (57.7)	2335 (59.2)	140 (36.0)	241 (56.6)	249 (66.8)	
Unknown	236 (4.6)	189 (4.8)	21 (5.4)	16 (3.8)	< 11 (< 2.9)	
Census tract poverty Indicator						<0.001
0 to < 5% poverty	1386 (27.0)	1183 (30.0)	> 15 (> 3.9)	> 62 (>14.6)	110 (29.5)	
5 to < 10% poverty	1459 (28.4)	1208 (30.6)	48 (12.3)	83 (19.5)	120 (32.2)	
10 to < 20% poverty	1426 (27.8)	1071 (27.1)	106 (27.2)	150 (35.2)	99 (26.5)	
20 to 100% poverty	834 (16.2)	463 (11.7)	209 (53.7)	120 (28.2)	> 33 (> 8.8)	
Missing	30 (0.6)	22 (0.6)	< 11 (< 2.8)	< 11 (< 2.6)	< 11 (< 2.9)	
Education level						<0.001
High school or less	2790 (54.3)	2000 (50.7)	263 (67.6)	316 (74.2)	211 (56.6)	
At least some college and higher	1926 (37.5)	1644 (41.7)	79 (20.3)	79 (18.5)	124 (33.2)	
Missing	419 (8.2)	303 (7.7)	47 (12.1)	31 (7.3)	38 (10.2)	
Medicare type						<0.001
MA PDP	1621 (31.6)	1083 (27.4)	181 (46.5)	192 (45.1)	165 (44.2)	
MA only	917 (17.9)	734 (18.6)	52 (13.4)	74 (17.4)	57 (15.3)	
FFS PDP	1029 (20.0)	828 (21.0)	65 (16.7)	68 (16.0)	68 (18.2)	
FFS only	1352 (26.3)	1156 (29.3)	65 (16.7)	77 (18.1)	54 (14.5)	
MA PPO	216 (4.2)	146 (3.7)	26 (6.7)	15 (3.5)	29 (7.8)	
Months from DX to survey						
(mean/SD)	48.6 (±34.3)	49.2 (±34.7)	44.5 (±32.1)	47.0 (±32.2)	48.3 (±34.8)	0.12
Stage at diagnosis						
0	550 (10.7)	422 (10.7)	48 (12.3)	34 (8.0)	> 45 (> 12.1)	0.025
I	1658 (32.3)	1262 (32.0)	130 (33.4)	141 (33.1)	125 (33.5)	
II and III	2627 (51.2)	2035 (51.6)	174 (44.7)	230 (54.0)	188 (50.4)	
IV	203 (4.0)	157 (4.0)	25 (6.4)	11 (2.6)	< 11 (< 2.9)	
Radiation Yes						
Yes	459 (8.9)	345 (8.7)	> 26 (> 6.7)	> 35 (> 8.2)	> 27 (> 7.2)	0.75
No	4623 (90.0)	3556 (90.1)	352 (90.5)	380 (89.2)	335 (89.8)	
Missing	53 (1.0)	46 (1.2)	< 11 (< 2.8)	< 11 (< 2.6)	< 11 (< 2.9)	
Surgery						0.27
Yes	4930 (96.0)	3804 (96.4)	360 (92.5)	> 404 (>94.8)	> 351 (94.1)	
No	68 (1.3)	48 (1.2)	< 11 (< 2.8)	< 11 (< 2.6)	< 11 (< 2.9)	
Missing	137 (2.7)	95 (2.4)	> 18 (> 4.6)	11 (2.6)	11 (2.9)	
SEER region						<0.001
West	2657 (51.7)	1884 (47.7)	78 (20.1)	337 (79.1)	340 (91.2)	
Midwest	484 (9.4)	436 (11.0)	38 (9.8)	< 11 (< 2.6)	< 11 (< 2.9)	
Northeast	903 (17.6)	771 (19.5)	72 (18.5)	51 (12.0)	< 11 (< 2.9)	
South	1091 (21.2)	856 (21.7)	201 (51.7)	> 27 (> 6.3)	< 11 (< 2.9)	
Comorbidities						0.011
0	3372 (65.7)	2630 (66.6)	229 (58.9)	272 (63.8)	241 (64.6)	
1	1193 (23.2)	883 (22.4)	110 (28.3)	99 (23.2)	101 (27.1)	
2+	570 (11.1)	434 (11.0)	50 (12.9)	55 (12.9)	31 (8.3)	
Survey mode						<0.001
Mail	>871 (>17.0)	>607 (>15.4)	>103 (>26.5)	>96 (>22.5)	>32 (>8.6)	
Phone	4253 (82.8)	3329 (84.3)	275 (70.7)	319 (74.9)	330 (88.5)	
Missing	<11 (<0.2)	<11 (<0.3)	<11 (<2.8)	<11 (<2.6)	<11 (<2.9)	
Survey year						<0.001
(1996, 2005]	1097 (21.6)	885 (22.6)	63 (16.3)	89 (21.1)	60 (16.3)	
(2005, 2008]	1066 (21.0)	845 (21.6)	65 (16.8)	94 (22.3)	62 (16.8)	
(2008, 2010]	1264 (24.8)	911 (23.3)	140 (36.3)	109 (25.8)	104 (28.2)	
(2010, 2013]	1661 (32.6)	1270 (32.5)	118 (30.6)	130 (30.8)	143 (38.8)	

Note: ^1^Individual cell counts less than 11 have been suppressed for patient privacy; ^2^*n* (%); median (IQR); ^3^Pearson’s chi-squared test (for categorical variables); Kruskal-Wallis rank sum test (for continuous variables)

### Composite Scores and Ratings of Care

Overall composite scores, as well as scores stratified by demographic characteristics, are presented in Table 2. Of the composite scores, patients reported “getting all needed prescriptions” the highest (90.8), followed by “physician communication” (89.0), “getting all needed care” (88.0), and “getting care quickly” (84.1). We found that non-Hispanic Asian patient composite scores vary greatly, anywhere from 3 to 11 points lower than the overall mean of NHW, depending on the composite score category.

**Table 2:** Association between demographics and composite care scores and ratings^1^

	**Composite scores**							
	**Getting care quickly**		**Get needed care**		**Physician communication**		**Getting needed prescription drugs**	
No. analyzed, *N* (%)	3847		3798		3861		4337	
Overall	84.1 (23.3)		88.0 (19.9)		89.0 (16.9)		90.8 (20.1)	
	LSM (±SE)	*P*-value^2^	LSM (+SE)	*P*-value^2^	LSM (+SE)	*P*-value^2^	LSM (+SE)	*P*-value^2^
Race		<0.001		<0.001		0.005		<0.001
Non-Hispanic White	85.4 (22.2)		88.9 (18.9)		89.0 (16.9)		92.0 (18.3)	
Non-Hispanic Black	81.8 (24.8)		86.8 (21.9)		90.4 (15.3)		86.4 (25.2)	
Hispanic	83.1 (24.8)		87.0 (22.5)		90.1 (16.8)		87.1 (25.5)	
Non-Hispanic Asian	72.6 (28.4)		80.2 (23.6)		86.0 (18.5)		86.5 (24.5)	
Age at survey		0.20		0.24		0.34		0.92
(0,70]	85.1 (22.6)		89.9 (18.1)		89.4 (16.5)		89.5 (22.3)	
(70,75]	85.5 (21.9)		89.0 (19.2)		89.9 (16.1)		91.1 (19.7)	
(75,80]	84.7 (23.2)		87.4 (20.9)		89.5 (16.6)		90.7 (19.8)	
(80,85]	81.9 (25.1)		87.4 (19.4)		88.2 (17.7)		90.8 (20.2)	
(85,Inf]	84.4 (22.7)		87.5 (20.4)		88.4 (17.3)		91.1 (20.2)	
Missing								
Cancer stage		0.09		0.81		0.86		0.86
0	83.5 (24.3)		87.9 (18.7)		88.6 (17.3)		91.4 (19.2)	
I	83.5 (23.5)		87.3 (21.0)		89.0 (17.3)		91.0 (19.7)	
II/III	84.3 (23.3)		88.4 (19.3)		89.0 (16.8)		90.5 (20.7)	
IV	90.1 (16.7)		89.3 (19.7)		90.4 (15.3)		92.1 (19.1)	
Missing	81.7 (24.6)		86.4 (22.8)		88.0 (15.7)		91.4 (19.6)	
Census poverty		0.97		0.78		0.97		0.01
[0%, 5%)	84.4 (22.5)		87.6 (19.8)		88.7 (17.1)		92.0 (19.9)	
[5%, 10%)	84.3 (23.2)		88.2 (19.1)		88.8 (17.2)		92.2 (19.1)	
[10%, 20%)	84.4 (23.4)		88.3 (19.4)		89.2 (17.0)		90.4 (20.0)	
[20%, 100%]	83.3 (24.1)		87.8 (22.2)		89.5 (15.9)		88.9 (22.3)	
Missing	75.7 (34.8)		87.3 (18.2)		91.0 (18.4)		91.5 (18.9)	
Comorbidities		0.49		<0.001		0.30		0.001
0	84.3 (23.5)		88.8 (19.9)		88.6 (17.2)		91.2 (20.5)	
1	83.4 (23.6)		86.4 (19.5)		90.0 (15.9)		90.0 (20.0)	
2+	84.8 (21.3)		86.3 (19.4)		89.3 (17.0)		90.6 (18.3)	
Education		0.82		0.01		0.82		0.82
HS or less	83.8 (24.2)		88.6 (20.1)		88.9 (17.4)		90.4 (21.2)	
Some college +	85.0 (21.6)		87.5 (19.5)		89.2 (16.1)		91.6 (18.1)	
Missing	82.2 (25.1)		86.3 (19.8)		88.9 (17.4)		89.4 (22.0)	
Gender		0.13		0.49		0.20		0.96
Female	84.8 (22.9)		87.7 (20.2)		89.1 (17.5)		90.9 (19.9)	
Male	83.4 (23.7)		88.4 (19.5)		88.9 (16.4)		90.8 (20.4)	
Marital status		0.66		0.91		0.99		0.30
Unmarried	84.4 (23.5)		88.0 (20.2)		88.8 (17.4)		90.0 (21.4)	
Married	83.9 (23.2)		88.1 (19.8)		89.1 (16.7)		91.2 (19.5)	
Missing	84.9 (22.1)		86.9 (18.2)		90.0 (16.0)		92.6 (17.1)	
Medicare status		0.006		0.01		<0.001		<0.001
MA PDP	82.0 (25.0)		85.9 (20.6)		89.7 (16.1)		90.0 (20.1)	
MA only	85.3 (21.6)		90.0 (20.4)		87.5 (17.6)		91.8 (20.9)	
FFS PDP	83.9 (23.5)		86.1 (20.0)		90.0 (16.7)		89.3 (20.6)	
FFS only	85.8 (21.9)		89.3 (18.9)		88.5 (17.7)		92.6 (19.1)	
MA PPO	85.8 (23.2)		90.7 (14.9)		90.4 (15.1)		89.9 (20.7)	
SEER region		0.001		< 0.001		0.006		0.11
West	83.0 (23.5)		86.9 (20.6)		88.3 (17.4)		91.0 (20.0)	
Mid-West	87.3 (21.1)		91.3 (16.7)		90.6 (15.9)		92.3 (18.6)	
Northeast	84.3 (23.5)		87.6 (20.3)		88.6 (17.6)		90.1 (21.6)	
South	85.5 (23.2)		89.7 (18.5)		90.4 (15.3)		90.3 (19.9)	
Radiation		0.91		0.91		0.91		0.91
Yes	85.3 (21.7)		87.7 (19.6)		88.7 (16.7)		90.3 (21.2)	
No	83.9 (23.5)		87.9 (19.9)		89.0 (16.9)		90.8 (20.1)	
Missing	91.3 (16.2)		96.2 (12.4)		92.1 (12.9)		94.1 (17.8)	
Surgery		0.93		0.93		0.93		0.93
Yes	84.1 (23.3)		88.1 (19.7)		89.0 (16.9)		90.9 (20.1)	
No	82.0 (25.4)		84.3 (24.9)		87.8 (15.7)		89.7 (20.3)	
Missing	85.7 (21.1)		85.2 (22.9)		88.6 (16.9)		89.1 (22.2)	
Survey mode		0.06		0.70		0.70		0.05
Mail	80.0 (28.6)		85.7 (24.3		89.1 (17.6)		87.6 (24.6)	
Phone	84.9 (22.2)		88.3 (19.0)		89.0 (16.8)		91.4 (19.1)	
	**Ratings of care**							
	**Rating primary physician**		**Rating for specialist**		**Rating for health care**		**Rating for health plan**	
No. analyzed, *N* (%)	3913		2903		4081		4553	
Overall	9.0 (1.5)		9.0 (1.6)		8.7 (1.7)		8.7 (1.8)	
	LSM (± SE)	*P*-value	LSM (± SE)	*P*-value	LSM (± SE)	*P*-value	LSM (+ SE)	*P*-value
Race		N/A		N/A		N/A		N/A
Non-Hispanic White	N/A		N/A		N/A		N/A	
Non-Hispanic Black	N/A		N/A		N/A		N/A	
Hispanic	N/A		N/A		N/A		N/A	
Non-Hispanic Asian	N/A		N/A		N/A		N/A	
Age at survey		0.793		0.92		0.592		0.0064
(0,70]	9.0 (1.5)		9.1 (1.31)		8.9 (1.4)		8.6 (1.7)	
(70,75]	9.0 (1.5)		8.98 (1.62)		8.7 (1.7)		8.5 (1.9)	
(75,80]	9.1 (1.5)		9 (1.61)		8.7 (1.7)		8.7 (1.7)	
(80,85]	9.0 (1.6)		8.96 (1.71)		8.6 (1.8)		8.7 (1.7)	
(85,Inf]	9.0 (1.6)		8.93 (1.62)		8.6 (1.8)		8.8 (1.7)	
Missing								
Cancer stage		0.864		0.318		0.318		0.0576
0	9.0 (1.4)		8.94 (1.55)		8.6 (1.6)		8.5 (1.8)	
I	9.0 (1.6)		8.92 (1.67)		8.7 (1.8)		8.6 (1.8)	
II/III	9.0 (1.5)		9.02 (1.6)		8.7 (1.6)		8.7 (1.7)	
IV	9.0 (1.6)		9.07 (1.61)		8.8 (1.8)		8.8 (1.6)	
Missing	8.9 (1.5)		8.91 (1.52)		8.4 (2.2)		8.6 (2.1)	
Census poverty		0.0376		0.999		0.674		0.0008
[0%, 5%)	8.9 (1.6)		9.04 (1.44)		8.7 (1.6)		8.6 (1.7)	
[5%, 10%)	8.9 (1.6)		9 (1.53)		8.7 (1.7)		8.6 (1.8)	
[10%, 20%)	9.0 (1.6)		8.92 (1.78)		8.7 (1.8)		8.7 (1.8)	
[20%, 100%]	9.2 (1.3)		8.93 (1.78)		8.7 (1.8)		8.8 (1.8)	
Missing	9.4 (1.2)		9.24 (1.35)		9.0 (1.5)		8.7 (2.1)	
Comorbidities		0.767		0.30		< 0.001		0.126
0	9.0 (1.6)		8.99 (1.65)		8.8 (1.7)		8.7 (1.7)	
1	9.1 (1.4)		8.95 (1.57)		8.6 (1.8)		8.6 (1.8)	
2+	9.0 (1.5)		8.98 (1.49)		8.5 (1.8)		8.5 (1.8)	
Education		0.0107		0.127		0.0242		< 0.001
HS or less	9.0 (1.6)		8.95 (1.77)		8.7 (1.8)		8.8 (1.8)	
Some college +	9.0 (1.4)		9 (1.46)		8.7 (1.6)		8.5 (1.7)	
Missing	9.0 (1.6)		9.11 (1.23)		8.4 (2.1)		8.6 (1.7)	
Gender		0.0004		0.174		0.00213		< 0.001
Female	9.0 (1.6)		8.99 (1.66)		8.7 (1.7)		8.7 (1.7)	
Male	9.0 (1.5)		8.98 (1.56)		8.6 (1.7)		8.6 (1.8)	
Marital status		0.461		0.905		0.818		0.298
Unmarried	9.0 (1.6)		8.95 (1.69)		8.7 (1.8)		8.7 (1.8)	
Married	9.0 (1.5)		9.01 (1.55)		8.7 (1.7)		8.6 (1.7)	
Missing	9.0 (1.6)		8.8 (1.85)		8.7 (1.6)		8.5 (1.8)	
Medicare status		0.08		0.18		0.53		0.10
MA PDP	9.0 (1.5)		8.94 (1.57)		8.6 (1.8)		8.7 (1.7)	
MA only	8.9 (1.6)		8.99 (1.63)		8.8 (1.6)		8.7 (1.7)	
FFS PDP	9.1 (1.4)		8.92 (1.58)		8.6 (1.8)		8.7 (1.8)	
FFS only	9.0 (1.5)		9.04 (1.61)		8.8 (1.6)		8.7 (1.8)	
MA PPO	9.0 (1.5)		8.98 (1.94)		8.7 (1.7)		8.4 (2.0)	
SEER region		< 0.001		0.04		0.006		0.02
West	8.9 (1.6)		8.93 (1.65)		8.7 (1.7)		8.6 (1.7)	
Mid-West	9.1 (1.5)		9.12 (1.44)		8.9 (1.6)		8.8 (1.7)	
Northeast	8.9 (1.6)		8.95 (1.57)		8.6 (1.8)		8.6 (1.8)	
South	9.2 (1.3)		9.07 (1.62)		8.7 (1.8)		8.8 (1.8)	
Radiation		0.57		0.91		0.91		0.91
Yes	8.9 (1.6)		8.92 (1.84)		8.6 (1.9)		8.7 (1.8)	
No	9.0 (1.5)		8.99 (1.58)		8.7 (1.7)		8.7 (1.8)	
Missing	9.4 (0.9)		9.12 (2.15)		8.8 (1.8)		9.0 (1.6)	
Surgery		0.93		0.93		0.93		0.93
Yes	9.0 (1.5)		8.98 (1.61)		8.7 (1.7)		8.7 (1.7)	
No	8.8 (1.6)		8.71 (2.14)		8.7 (1.8)		8.6 (1.9)	
Missing	8.7 (2.0)		9.18 (1.35)		8.3 (2.4)		8.4 (2.0)	
Survey mode		0.53		0.75		0.05		0.15
Mail	8.9 (1.8)		8.88 (1.85)		8.5 (1.9)		8.7 (1.9)	
Phone	9.0 (1.5)		8.99 (1.58)		8.7 (1.7)		8.7 (1.7)	

^1^Analysis of race/ethnic differences on global ratings is not provided due to literature support that it is invalid to make these comparisons; ^2^ adjusted *p*-values were computed from 2-sided Wilcoxon rank sum test (when there are only two categories) or the Kruskal-Wallis test (when there are more than two categories) using the Benjamini-Hochberg method

### Regression Analyses

Adjusted regression analyses are presented in Table 3. Each score/rating represents a separate model, but all covariates are included simultaneously. These models show the presence of ethnic/racial disparities in the composite score. Namely, adjusting for all covariates, non-Hispanic Asians had lower scores of physician communication (*B* = − 2.72, *p* = 0.01), getting care quickly (*B* = − 11.81, *p* < 0.001), getting needed care (*B* = − 7.84, p < 0.001), and getting needed prescriptions (*B* = − 5.66, *p* < 0.001). In fact, all non-White ethnic/racial groups had trouble getting needed prescriptions, including NHB (*B* = − 4.32, *p* < 0.001) and Hispanics (*B* = − 4.34, *p* < 0.001). NHB additionally had trouble getting care quickly compared to NHW (*B* = − 3.44, *p* = 0.03).

**Table 3:** Regression coefficients for multivariable regressions of composite scores on patient sociodemographic, health, and cancer history characteristics

	Getting care quickly	Getting needed care	Physician communication	Get needed prescription drugs
	3847	3798	3861	4337
Race/ethnicity	-	-	-	-
Non-Hispanic White				
Non-Hispanic Asian	−11.81 (−14.91, −8.71)***	− 7.84 (− 10.45, − 5.23)***	− 2.72 (− 4.9, -0.54)*	− 5.66 (− 8.11, − 3.21)***
Non-Hispanic Black	− 3.44 (−6.6, −0.28)*	− 2.22 (− 4.94, 0.5)	0.62 (− 1.59, 2.83)	− 4.32 (− 6.81, − 1.83)***
Hispanic	−0.81 (−3.57, 1.95)	− 0.97 (− 3.36, 1.42)	1.42 (− 0.6, 3.44)	− 4.34 (− 6.63, − 2.05)***
Gender	-	-	-	-
Female				
Male	−1.82 (−3.39, −0.25)*	0.81 (−0.52, 2.14)	−0.19 (−1.35, 0.97)	−0.66 (−1.93, 0.61)
Age at survey				
	−0.54 (−1.72, 0.64)	−0.72 (−1.72, 0.28)	−0.69 (−1.55, 0.17)	−0.3 (−1.26, 0.66)
Marital status at diagnosis	-	-	-	-
Unmarried				
Married	−0.26 (−1.95, 1.43)	−0.47 (−1.9, 0.96)	0.3 (−0.92, 1.52)	0.85 (-0.5, 2.2)
Education level	-	-	-	-
H.S. or less				
Some				
College +	1.14 (−0.47, 2.75)	−1.12 (−2.49, 0.25)	0.64 (−0.54, 1.82)	0.17 (−1.14, 1.48)
Census tract poverty indicator				
[0%, 5%)	-	-	-	-
[10%, 20%)	0.17 (−1.87, 2.21)	0.73 (−1.01, 2.47)	0.09 (−1.42, 1.6)	−0.1 (−1.79, 1.59)
[20%, 100%]	−0.27 (−2.78, 2.24)	0.42 (−1.78, 2.62)	0.01 (−1.85, 1.87)	−0.41 (−2.47, 1.65)
[5%, 10%)	−0.09 (−2.05, 1.87)	0.44 (−1.25, 2.13)	0.03 (−1.42, 1.48)	1.19 (−0.44, 2.82)
Months from DX to Svy	-	-	-	-
	−0.86 (−1.57, −0.15)*	−0.11 (−0.74, 0.52)	0.24 (−0.29, 0.77)	0.49 (−0.08, 1.06)+
Cancer stage	-	-	-	-
0				
I	−0.46 (−3.07, 2.15)	−0.98 (−3.23, 1.27)	0.45 (−1.45, 2.35)	−0.33 (−2.45, 1.79)
II/III	0.11 (−2.42, 2.64)	−0.07 (−2.25, 2.11)	0.71 (−1.13, 2.55)	−0.89 (−2.93, 1.15)
IV	5.26 (1.05, 9.47)*	0.57 (−3, 4.14)	2.23 (−1.04, 5.5)	1.18 (−2.39, 4.75)
Radiation	-	-	-	-
No				
Yes	1.55 (−1.04, 4.14)	−0.38 (−2.61, 1.85)	−0.45 (−2.39, 1.49)	−0.01 (− 2.15, 2.13)
Surgery	-	-	-	-
No				
Yes	1.16 (−5.72, 8.04)	3.13 (−2.67, 8.93)	0.51 (−4.76, 5.78)	1.7 (−3.83, 7.23)
SEER region	-	-	-	-
West				
Mid-West	2.76 (0.06, 5.46)*	3.32 (1.01, 5.63)**	2.15 (0.15, 4.15)*	0.49 (−1.71, 2.69)
Northeast	0.15 (−2.05, 2.35)	−0.09 (−1.97, 1.79)	0.26 (−1.35, 1.87)	−1.56 (−3.36, 0.24)
South	1.59 (−0.47, 3.65)	2.01 (0.23, 3.79)*	1.62 (0.09, 3.15)*	−0.78 (−2.49, 0.93)
Medicare type	-	-	-	-
MA PDP				
FFS only	2.46 (0.54, 4.38)*	2.41 (0.72, 4.1)**	−1.39 (−2.82, 0.04)	1.73 (0.1, 3.36)*
FFS PDP	0.92 (−1.29, 3.13)	−0.46 (−2.44, 1.52)	0.05 (−1.6, 1.7)	−0.97 (−2.69, 0.75)
MA only	2.02 (−0.27, 4.31)	3.01 (1.09, 4.93)**	−1.83 (−3.5, −0.16)*	1.3 (−0.62, 3.22)
MA PPO	3.79 (−0.01, 7.59)	4.67 (1.34, 8)**	0.46 (−2.28, 3.2)	−0.09 (−3.21, 3.03)
Comorbidities	-	-	-	-
0				
1	0.14 (−1.66, 1.94)	−1.56 (−3.17, 0.05)	0.86 (−0.47, 2.19)	−0.78 (−2.25, 0.69)
2+	1.37 (−0.94, 3.68)	−1.88 (−3.92, 0.16)	0.16 (−1.56, 1.88)	−0.26 (−2.16, 1.64)
Survey mode	-	-	-	-
Mail				
Phone	5.1 (2.98, 7.22)***	3.07 (1.23, 4.91)**	0.24 (−1.29, 1.77)	3.19 (1.52, 4.86)***

*p < 0.05, **p < 0.01, ***p < 0.001

### Survival Analysis

Unadjusted Cox proportional hazards analysis showed that between 2000 and 2013, NHB and non-Hispanic Asians diagnosed with colorectal cancer were at greater risk of all-cause mortality than NHW (H.R. = 1.24 and 1.23, respectively, *p*’s < 0.001). Mortality for Hispanics did not differ from Whites during this time period (OR = 1.09, *p* = 0.19).

**Fig. 1:**
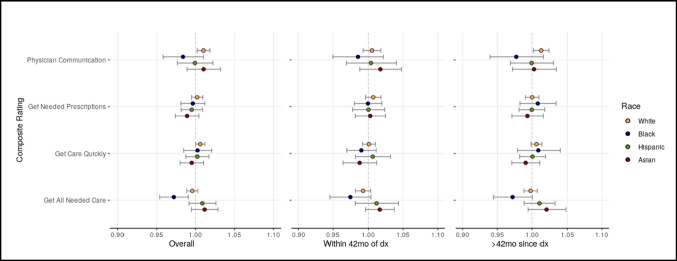
Race-specific hazard ratios for survival, adjusting for sex, survey age, marital status, region, poverty, comorbidities, Medicare type, cancer type, and dx to survey date. Restricted to individuals with colorectal cancer. Customer service could not be computed; sample size too low for those > 42 m of dx. Hazard ratios are for a 3-unit increase in composite score

Figure 1 depicts the race-specific hazard ratios for the effect of composite scores on mortality. Composite scores generally did not affect mortality, though a 3-unit increase in getting all needed care was associated with 0.97 times the hazard of mortality for NHB (*p* = 0.003). Further, to disentangle the effect of recency of survey completion affecting the race-by-score interaction on mortality, analyses were stratified by time from diagnosis to survey completion at the median (42 months). This analysis showed no significant effect of composite scores on mortality for any ethnic/racial group.

## Discussion

In this population-based sample of individuals diagnosed with colorectal cancer, we found that non-Hispanic Black patients, Hispanics, and non-Hispanic Asians had significantly lower scores with aspects of their health care experiences compared to non-Hispanic White patients. The scores of patient experiences with care were associated with the risk of death but only for NHB colorectal cancer patients. Specifically, NHB patients with a three-point increase in their composite score of getting all their needed care were associated with a lower risk of death, which was not found among NHW patients.

Overall, our results were consistent with previous literature that demonstrates non-Hispanic Asian patients report poorer patient experiences, including lower composite scores of physician communication, customer service, getting care quickly, getting needed care, and getting needed prescriptions compared to NHW patients [9, 17, 29–33]. Similarly, our findings were consistent with prior literature that NHB scores for getting needed prescriptions and getting care quickly were lower than NHW patients [9, 32, 34–36]. Furthermore, we found that, consistent with our previous work, Hispanic patients reported a lower score for getting needed care, getting care quickly, and getting needed prescriptions compared to NHW patients [9, 32]. Specifically, our results that NHB and Hispanic patients with CRC report lower CAHPS measure scores than NHW patients are similar to other studies of Medicare beneficiaries older than 65 years with breast, prostate, colorectal, and lung cancers [9, 22, 32, 37]. In addition, the consistency in our findings with the previous literature demonstrates that across various studies and populations, ethnic minorities have lower ratings of healthcare, and in many cases, these relationships persist even after controlling for other demographic and clinical characteristics. Thus, these associations suggest that there may be other factors that need to be further explore, for example, to fully understand these racial/ethnic disparities, we may need to incorporate other cultural influences, medical mistrust, medical discrimination, etc.

Further, we found that better composite scores for getting all needed care among NHB patients were associated with decreased risk of mortality. A SEER-CHAPS study found that better global ratings of overall health care and primary care providers were associated with higher odds of adherence to surveillance procedures such as receipt of a colonoscopy, CT imaging, CEA tests, and office visits [11]. Our findings, along with Mollica and colleagues’ findings, suggest that better patient experiences with overall health care and primary care providers may encourage adherence to cancer surveillance and may be more critical for survival among Black colorectal cancer patients. In addition, these results are similar to our prior findings among NHB lung cancer survivors, where patients with better experiences with getting care were associated with lower mortality risk [22].

### Strengths and Limitations

We constructed a large retrospective cohort study of patients over the age of 65 on Medicare Advantage and Medicare fee-for-service insurance with complete information on sociodemographic, clinical, and cancer prognostic factors. Thus, our study is comprehensive and geographically diverse. To the best of our knowledge, this is the first population-based study to examine racial/ethnic disparities in patient experiences with health care and mortality among colorectal cancer patients. The linkage of detailed cancer registry data includes the stage at diagnosis, tumor characteristics, vital statistics, and demographic information. The patient surveys on patient experiences allowed us to examine the association of patient experiences with cancer outcomes using the SEER-CAHPS dataset.

Our study had a few limitations. First, the assessment of patient experiences is cross-sectional, and patient experiences may change over time. However, we did stratify the analysis by the median time from diagnosis to survey completion and found no significant association. Second, questions on patient experiences with care were related to overall medical care rather than care explicitly pertaining to colorectal cancer treatment and surveillance. Thus, our study results suggest that overall care for colorectal cancer survivors may be just as important as patient experiences with cancer treatment. Third, our analysis was limited to patients aged 65 and older enrolled in Medicare fee-for-service and Medicare advantage diagnosed with cancer within the SEER-registry regions. Finally, to be included in the study, individuals had to complete a CAHPS survey after diagnosis. Therefore, we may have fewer individuals in poorer health or at higher risk of death than all colorectal cancer patients.

## Implications

Our study findings highlight the need to understand the racial/ethnic-specific determinants of CAHPS measures. Without such information, current efforts by healthcare practices to improve CAHPS measures may exacerbate disparities in cancer outcomes if CAHPS measures do not address the unique experiences for minority patients with cancer. Further studies among patients over the age of 65 with other health insurance or that are not insured need to be conducted in order to understand other unique experiences.

## Conclusion

In summary, our findings highlight the potential importance in understanding why racial disparities exist in medical experiences among individuals diagnosed with colorectal cancer which greatly impact CRC mortality. Specifically we found that NHB, Hispanics, or non-Hispanic Asians, report who report worse patient experiences with care compared to NHW patients even after controlling for various demographic and clinical factors. Facilitation of this knowledge may improve awareness of what factors influence ratings of patients’ experiences of medical care that differ among racial/ethnic groups and whether these experiences influence engagement in subsequent healthcare and mortality. Future studies that account for these factors may help with the development of culturally tailored intervention for ethnic minorities, which may look similar or different among NHB, Hispanics, and non-Hispanic Asians.
